# Exploration of the Mouth

**Published:** 1868-08

**Authors:** W. H. Waite

**Affiliations:** Liverpool, England.


### ARTICLE VII.

### *Exploration of the Mouth.*

By W. H. Waite, D. D. S., Liverpool, England.

In order to make a thorough examination as to the condition of a patient's mouth, it is desirable that the seat of the operating chair should be raised, so as to bring the mouth within easy command of the operator's eye, without necessitating any awkward or uncomfortable attitude. The patient, being seated, may be directed to rinse the mouth with tepid water, in order to remove any fragments of food, mucus, etc., which may be lying between the teeth. Then, defending the lip by a napkin, a probe is taken, and exploration commenced upon the right dens sapientia, proceeding around the upper jaw to the left wisdom tooth, then descending to the lower of the same side, return to the right lower wisdom teeth. The points of each tooth most liable to the attack of disease may be thus enumerated:

3rd molars. The disto-buccal angle, and masticating surface.

2nd molars. Masticating and buccal surfaces.

1st molars. Masticating and mesial surfaces.

2nd bicuspids. Distal, and often mesial surfaces.

1st bicuspids. Mesial, and sometimes distal surfaces.

Canines. Approximal, specially distal surfaces.

Laterals. Approximal surfaces, and lingual rarely.

Centrals. Approximal and labial surfaces.

These may be called the highways of disease, but careful examination frequently discovers various by-paths in divers directions.

Having ascertained the actual state of each tooth, it is well to acquaint the patient at once with the result, and at the same time indicate the treatment required. This done, there are just two or three things to be observed, viz :

1st. Not to make more than a *proximate* estimate of the cost.

2nd. Never to promise *positively* any result in doubtful cases.

3rd. Promise to do the best in our power.

4th. Never to find fault with other persons' work.

5th. Always to treat patient's opinion with respect, at the same time insisting on the maintenance of our own.

6th and lastly. Always to do to our patients as we would be done by.

10. Oxford Street.—*Canada Journal of Dental Science.*

